# Mobile-Genetic-Element-Encoded Hypertolerance to Copper Protects Staphylococcus aureus from Killing by Host Phagocytes

**DOI:** 10.1128/mBio.00550-18

**Published:** 2018-10-16

**Authors:** Marta Zapotoczna, Gustavo P. Riboldi, Ahmed M. Moustafa, Elizabeth Dickson, Apurva Narechania, Julie A. Morrissey, Paul J. Planet, Matthew T. G. Holden, Kevin J. Waldron, Joan A. Geoghegan

**Affiliations:** a Department of Microbiology, Moyne Institute of Preventive Medicine, School of Genetics and Microbiology, Trinity College Dublin, Dublin, Ireland; b Institute for Cell & Molecular Biosciences, Faculty of Medical Sciences, Newcastle University, Newcastle upon Tyne, United Kingdom; c Pediatric Infectious Disease Division, Children’s Hospital of Philadelphia, Abramson Pediatric Research Center, University of Pennsylvania, Philadelphia, Pennsylvania, USA; d Scottish MRSA Reference Service, Scottish Microbiology Reference Laboratories, Glasgow, United Kingdom; e American Museum of Natural History, New York, New York, USA; f Department of Genetics, University of Leicester, Leicester, United Kingdom; g School of Medicine, University of St. Andrews, St. Andrews, United Kingdom; New York University School of Medicine

**Keywords:** MRSA, P-type ATPase, *Staphylococcus aureus*, copper tolerance, macrophages, metals, mobile genetic elements, multicopper oxidase

## Abstract

Pathogens are exposed to toxic levels of copper during infection, and copper tolerance may be a general virulence mechanism used by bacteria to resist host defenses. In support of this, inactivation of copper exporter genes has been found to reduce the virulence of bacterial pathogens *in vivo*. Here we investigate the role of copper hypertolerance in methicillin-resistant Staphylococcus aureus (MRSA). We show that a copper hypertolerance operon (*copB-mco*), carried on a mobile genetic element (MGE), is prevalent in a collection of invasive S. aureus strains and more widely among clonal complex 22, 30, and 398 strains. The *copB* and *mco* genes encode a copper efflux pump and a multicopper oxidase, respectively. Isogenic mutants lacking *copB* or *mco* had impaired growth in subinhibitory concentrations of copper. Transfer of a *copB-mco-*carrying plasmid to a naive clinical isolate resulted in a gain of copper hypertolerance and enhanced bacterial survival inside primed macrophages. The *copB* and *mco* genes were upregulated within infected macrophages, and their expression was dependent on the copper-sensitive operon repressor CsoR. Isogenic *copB* and *mco* mutants were impaired in their ability to persist intracellularly in macrophages and were less resistant to phagocytic killing in human blood than the parent strain. The importance of copper-regulated genes in resistance to phagocytic killing was further elaborated using mutants expressing a copper-insensitive variant of CsoR. Our findings suggest that the gain of mobile genetic elements carrying copper hypertolerance genes contributes to the evolution of virulent strains of S. aureus that are better equipped to resist killing by host immune cells.

## Abstract (precis)

Methicillin-resistant Staphylococcus aureus (MRSA) poses a substantial threat to human health worldwide and evolves rapidly by acquiring mobile genetic elements, such as plasmids. Here we investigate how the *copB*-*mco* copper hypertolerance operon carried on a mobile genetic element contributes to the virulence potential of clinical isolates of MRSA. Copper is a key component of innate immune bactericidal defenses. Here we show that copper hypertolerance genes enhance the survival of S. aureus inside primed macrophages and in whole human blood. The *copB* and *mco* genes are carried by clinical isolates responsible for invasive infections across Europe, and more broadly among three successful clonal lineages of S. aureus. Our findings show that a gain of copper hypertolerance genes increases the resistance of MRSA to phagocytic killing by host immune cells and imply that acquisition of this mobile genetic element can contribute to the success of MRSA.

## INTRODUCTION

Methicillin-resistant Staphylococcus aureus (MRSA) is a major problem for animal and human health and is considered a global high-priority pathogen by the World Health Organization (1). One reason why MRSA continues to be a problem is that it evolves rapidly by acquiring mobile genetic elements (MGEs) such as plasmids. Many successful contemporary clones of MRSA carry copper tolerance genes located on MGEs (2–6), but the contribution of copper hypertolerance to the fitness and virulence of S. aureus has not yet been studied.

Copper is a key component of innate immune bactericidal defenses, and macrophages use copper to kill intracellular bacteria by actively importing it into the phagosome (7–10). Eukaryotic copper transport is facilitated by CTR1-mediated import into the cell and ATP7a-dependent transport into the phagolysosome (7, 11). Under aerobic conditions, excess copper is proposed to catalyze the production of hydroxyl radicals via the Fenton and Haber-Weiss reactions, which may cause oxidative damage to macromolecules due to their high redox potential. Copper toxicity (under all conditions or perhaps only anoxic conditions) involves the formation of adventitious Cu(I)-thiolate bonds, thus damaging enzymes that functionally require free cysteines or disulfide bonds, such as iron sulfur cluster proteins (12, 13). The toxic properties of copper are harnessed by host phagocytes, such as macrophages (11, 14). Infection signaling, which involves elevated levels of interferon gamma (IFN-γ) and a release of copper into the plasma, may trigger activation of macrophages and increased import of copper, which enhances killing of phagocytosed bacteria (7, 10, 15).

Pathogens have evolved mechanisms to counteract copper toxicity, mainly by limiting the copper concentration in their cytoplasm through efflux or sequestration by copper metallochaperones, metallothioneins, or storage proteins (16). Almost all bacteria possess genes that confer copper tolerance, from environmental bacteria isolated from black shale in copper-rich exploration regions (17) to human pathogens. Inactivation of copper exporter genes has been shown *in vivo* to reduce the virulence of bacterial pathogens such as Mycobacterium tuberculosis (18), Streptococcus pneumoniae (19), Salmonella enterica (10), and Pseudomonas aeruginosa (20). In some cases, the virulence defect has been shown to be due to the inability of these pathogens to resist copper-mediated killing within the macrophage phagosome (10). Data accumulated so far suggest that copper tolerance may be a general mechanism of virulence in bacteria and that pathogens are exposed to toxic levels of copper during infection (10, 18, 19, 21).

**FIG 1:**
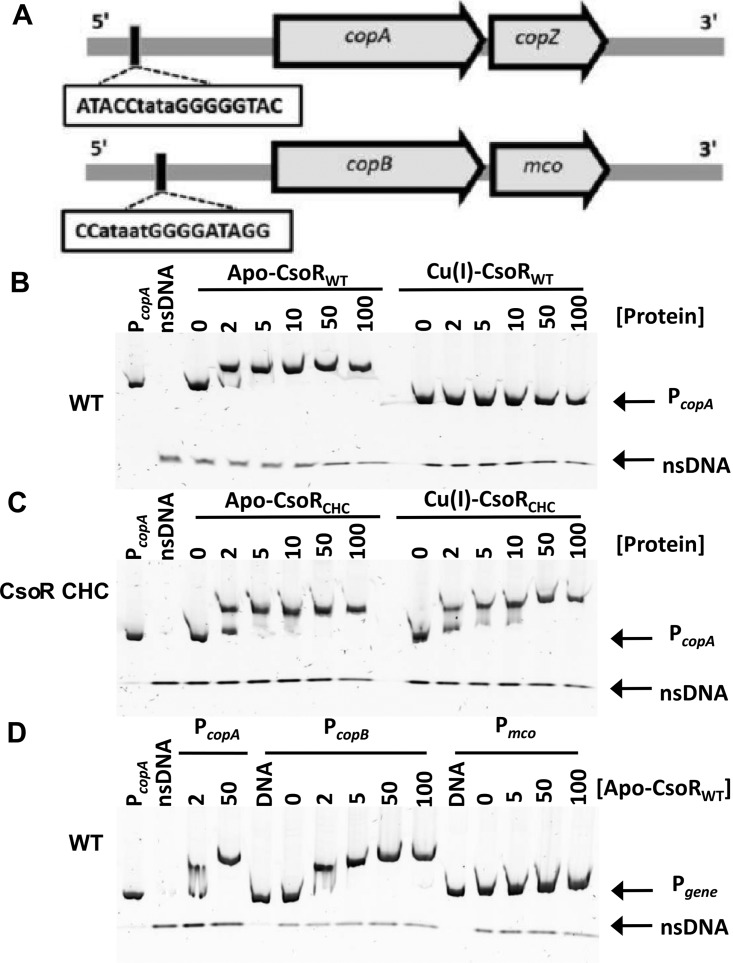
Electrophoretic mobility shift assay analysis of recombinant CsoR variants binding to the putative promoters upstream of *copA* (P*_copA_*), *copB* (P*_copB_*), and *mco* (P*_mco_*). (A) Schematic representation of the *copA*-*copZ* and *copB*-*mco* operons with putative promoter sequences. (B to D) Recombinant wild-type (WT) CsoR_WT_ or the CsoR_CHC_ mutant (CsoR C41A/H66A/C70A [CHC]) were purified and tested for binding to PCR products containing the DNA sequences (∼200 bp) upstream of the respective start codon, and a control DNA fragment of nonspecific DNA sequence (nsDNA). The concentrations of protein (in micromolar) are shown above the lanes. (B) Incubation of P*_copA_* DNA with wild-type apo-CsoR, but not Cu(I)-CsoR, retards the migration of P*_copA_*. (C) CsoR CHC retards migration of P*_copA_* in both the presence and absence of Cu(I). (D) CsoR retards migration of P*_copA_* and P*_copB_*, but not migration of P*_mco_*.

All S. aureus strains possess a conserved chromosomal operon, encoding the archetypal P_1B-1_-type ATPase copper transporter CopA and a copper metallochaperone CopZ, that confers low-level resistance to copper (Fig. 1A) (22). A copper hypertolerance operon (*copB*-*mco*) has been reported in some clinically relevant strains of S. aureus, carried either on a replicating plasmid or on a plasmid integrated into the chromosome (Fig. 1A) (2, 3, 5). The *copB* gene encodes a second copper-exporting P_1B-3_-type ATPase (CopB), and *mco* encodes a multicopper oxidase implicated in copper homeostasis and the oxidative stress response (23). A chromosomally encoded homolog of the Cu-sensitive operon repressor (CsoR), first characterized in M. tuberculosis (24), was shown to control transcription of both operons in S. aureus (2).

Here we investigated the role of copper hypertolerance in S. aureus. We found that the *copB* and *mco* genes carried on a MGE improved bacterial growth under copper stress and enhanced bacterial survival within macrophages and in whole human blood. Expression of *copB* and *mco* was detected by intracellular bacteria isolated from macrophages, and CsoR was responsible for regulating expression of these genes *in vivo*. Finally, we determined the extent of carriage of *copB* and *mco* genes in a collection of invasive S. aureus isolates from European hospitals and in a more diverse collection of whole-genome-sequenced isolates from around the world.

## RESULTS

### The tolerance of S. aureus to copper is enhanced by the *copB*-*mco* operon.

The *copB*-*mco* copper hypertolerance operon is carried either on a replicating plasmid or on a plasmid integrated into the chromosome (2, 3, 5). The role of copper tolerance genes carried on MGEs in MRSA was studied using the *copB*-*mco* operon-carrying plasmid P2-hm (3), here named pSCBU. Plasmid pSCBU was previously found to be carried by a population of MRSA clonal complex 22 (CC22) bloodstream isolates from the United Kingdom and Ireland (3). For the purposes of this study, pSCBU was introduced into S. aureus CC22 strain 14-2533T (see Table S1 in the supplemental material). 14-2533T is a clinical isolate that is representative of the lineage where pSCBU was detected, but it does not carry the plasmid. This strain was chosen as a clean and receptive host to study plasmid-conferred phenotypes.

10.1128/mBio.00550-18.4 TABLE S1 Strains used in this study. Download Table S1, DOCX file, 0.02 MB. Copyright © 2018 Zapotoczna et al. 2018 Zapotoczna et al. This content is distributed under the terms of the Creative Commons Attribution 4.0 International license.

The level of copper tolerance in strain 14-2533T carrying *copB* and *mco* genes on the replicating plasmid pSCBU was determined by measuring the minimum inhibitory concentrations (MICs) to copper salts (Table 1). Copper tolerance was the highest in strain 14-2533T carrying the replicating plasmid pSCBU (11 mM CuCl_2_), whereas the same strain without pSCBU had a lower MIC (6 mM). The individual contributions of the *copB* and *mco* genes to copper tolerance were investigated by generating isogenic mutants carrying deletions in the copper tolerance genes on the plasmid pSCBU (pSCBUΔ*mco* and pSCBUΔ*copB* [Table S1 and Fig. S1]). Deletion of *mco* or *copB* resulted in a decrease in the MIC to 8 mM or 6 mM CuCl_2_, respectively (Table 1), indicating that these genes are the main contributors to pSCBU-mediated copper tolerance. Inactivation of the *copA* gene in the 14-2533T or 14-2533T(pSCBU) background did not change the MIC compared to the wild-type strain, suggesting that the *copB*-*mco* operon is the main mediator of copper hypertolerance (Table 1). In support of a role for *copB*-*mco* in overcoming copper toxicity, 14-2533T(pSCBU) contained less intracellular copper (83.89 µM per optical density at 600 nm [OD_600_] unit) than strain 14-2533T without the plasmid (174.53 µM per OD_600_ unit) following culture in tryptic soy broth (TSB) supplemented with a subinhibitory concentration of CuCl_2_ (4 mM). When cultured in TSB without added CuCl_2_, strain 14-2533T and 14-2533T(pSCBU) had similar intracellular copper contents (9.42 and 8.94 µM per OD_600_ unit of copper, respectively), as measured using inductively coupled plasma mass spectrometry. This suggests that copper efflux is an important mechanism of copper hypertolerance.

**TABLE 1:** Tolerance of S. aureus strains to metals^*a*^

Strain	*copAZ*^*b*^	*copB*^*b*^	*mco*^*b*^	MIC (mM) to Cu_2_SO_4_	*cadA*^*b*^	MIC to CdCl_2_^*c*^	MIC (mM) to ZnCl_2_^*c*^
14-2533T	ch	NE	NE	6	NE	20 µM	2
14-2533T *copA*::*spc*^*d*^	*copA* mutant	NE	NE	6	NE	20 µM	2
14-2533T(pSCBU)	ch	p	p	11	p	20 mM	20
14-2533T *copA*::*spc*(pSCBU)	*copA* mutant	p	p	11	p	20 mM	20
14-2533T(pSCBUΔ*copB*)	ch	Δ	p	6	p	20 mM	20
14-2533T(pSCBUΔ*mco*)	ch	p	Δ	8	p	20 mM	20
14-2533T CHC	ch	NE	NE	6	NE	20 µM	2
14-2533T CHC(pSCBU)	ch	p	p	10	p	20 mM	20
MRSA252	ch	ch	ch	8	NE	NT	NT
MRSA252 CHC	ch	ch	ch	5	NE	NT	NT

a MICs were determined by a microdilution method.

b The presence or absence and location of the *copAZ*, *copB*, *mco*, and *cadA* genes are shown as follows: ch, gene is incorporated into the chromosome; NE, gene is not carried by the strain; p, gene is carried on a replicating plasmid; Δ, gene has been deleted by mutation.

c NT, not tested.

d *spc*, spectinomycin resistance (*aad9*).

10.1128/mBio.00550-18.1 FIG S1 Schematic representations of mutations generated within the *copB*-*mco* operon of the S. aureus plasmid pSCBU. Deletions of either *copB* or *mco* were introduced using the pIMAY vector. Download FIG S1, TIF file, 0.2 MB. Copyright © 2018 Zapotoczna et al. 2018 Zapotoczna et al. This content is distributed under the terms of the Creative Commons Attribution 4.0 International license.

The pSCBU plasmid also encodes a cadmium efflux system (*cadA*), which is known to protect from intracellular accumulation of toxic Cd(II), Zn(II), and Co(II) (25). For a control, cadmium and zinc tolerance of the pSCBU variants was tested. We observed that pSCBU conferred resistance to cadmium and zinc (Table 1), which was unaffected by mutations in *copB* and *mco*, demonstrating that these genes do not influence tolerance to these metals.

### CsoR binds to *copB* promoter DNA in a copper-dependent manner.

The S. aureus copper-sensing transcriptional regulator (CsoR) was previously shown to negatively regulate both chromosomal and plasmid-carried copper tolerance genes (2). Dissociation of CsoR from the GC-rich palindromic promoter regions has been shown to occur at two copper-regulated operons (*copA-copZ* and *copB-mco*) in a copper-dependent manner (Fig. 1A) (24). The S. aureus CsoR protein shares 24% amino acid sequence identity with CsoR from Mycobacterium tuberculosis (2). In S. aureus, residues Cys^41^, His^66^, and Cys^70^ coordinate Cu(I) and CsoR with an alanine substitution at position 41 fails to dissociate from DNA in the presence of Cu(I) (26). Electrophoretic mobility shift assays (EMSAs) performed anaerobically with recombinant CsoR and an ∼250-bp DNA fragment representing the *copA* promoter (P*_copA_*) confirmed that the wild-type CsoR repressor bound specifically to the *copA* promoter, whereas anaerobic incubation with Cu(I) prevented association of CsoR with the promoter DNA (Fig. 1B). In contrast, a CsoR variant carrying C41A, H66A, and C70A substitutions (C41A/H66A/C70A substitutions [CHC variant herein]) remained bound to the *copA* promoter DNA despite the presence of copper (Fig. 1C), suggesting that it is unable to coordinate Cu(I) and therefore to undergo its copper-dependent allosteric conformational change. Thus, the CsoR CHC variant is insensitive to copper, and derepression of CsoR-regulated genes will not occur in cells expressing this variant.

Since *copB* and *mco* genes are responsible for hypertolerance to copper in S. aureus (Table 1), binding of CsoR to the *copB* and *mco* promoter regions was investigated. CsoR bound to DNA containing the sequence of the regions upstream of both *copA* and *copB* (P*_copB_*), but not that upstream of *mco* (P*_mco_*) (Fig. 1D), consistent with *copB* and *mco* being cotranscribed as part of an operon, under the regulatory control of CsoR through binding to P*_copB_*. There is no obvious CsoR binding sequence within the short intergenic region (14 bp) between *copB* and *mco* or in the 3ʹ sequence of *copB* (2).

### Copper hypertolerance enhances growth of S. aureus at subinhibitory concentrations of copper.

To study the role of the copper-sensitive operon repressor CsoR in copper tolerance, site-directed mutagenesis was conducted on the *csoR* genes on the chromosomes of strain 14-2533T (CC22) and the CC30 strain MRSA252 to introduce amino acid substitutions (C41A/H66A/C70A) that generate the copper-insensitive CsoR variant (CsoR CHC) (Table S1).

The MRSA252 strain carries a chromosomally integrated plasmid bearing the *copB*-*mco* operon and was more tolerant to copper (MIC of 8 mM) than its isogenic CsoR CHC mutant (5 mM) (Table 1), showing that CsoR represses the copper tolerance phenotype in strain MRSA252. In contrast, the CsoR CHC variant-expressing strain 14-2533T CHC(pSCBU) exhibited a MIC (10 mM) similar to the MIC of the parent strain 14-2553T(pSCBU) (11 mM) and an elevated MIC compared to the plasmid-negative 14-2553T host strain (6 mM). This may reflect the fact that CsoR does not fully repress *copB* (and by extension *mco*) expressed from multicopy plasmids, as shown previously by Baker et al. using a *csoR*-deficient mutant of strain ATCC 12600 (2).

**FIG 2:**
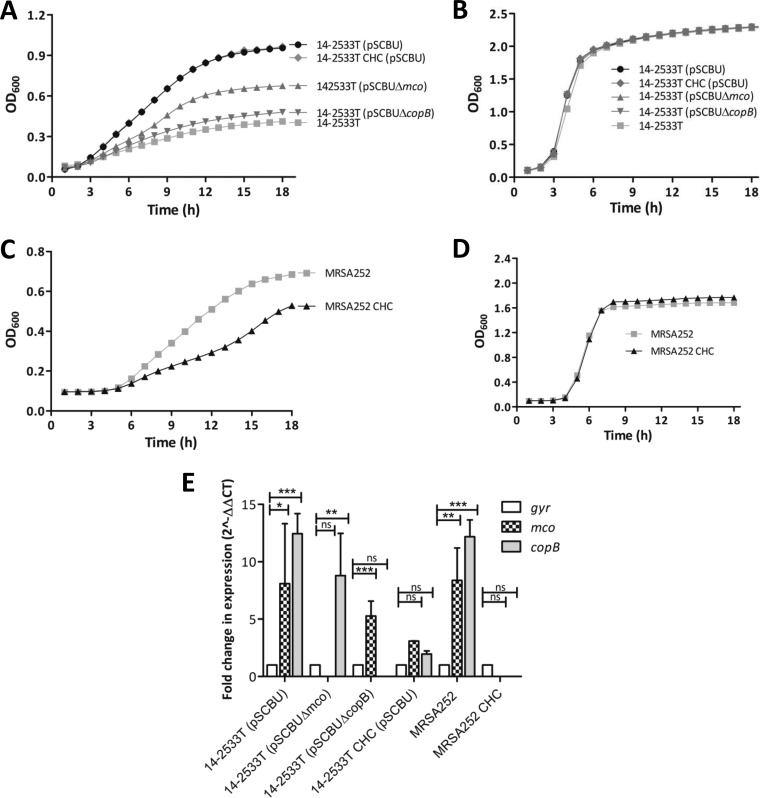
Enhanced growth in subinhibitory concentrations of copper chloride requires expression of copper tolerance genes. Growth of S. aureus 14-2533T and MRSA252 variants was measured in TSB supplemented with subinhibitory (4 mM) concentrations of copper chloride (A and C) or TSB broth alone (B and D). Growth curves representing data obtained from at least three independent experiments are presented. (E) Fold change in expression of *copB* and *mco* in S. aureus cultured in TSB versus TSB with copper chloride (4 mM). The ΔΔ*C_T_* method was used to determine the relative expression levels of the *copB* and *mco* genes normalized to *gyrB*. Values are means plus standard deviations (SD) (error bars) from three independent experiments, with statistical significance determined by analysis of variance (ANOVA). Values that are statistically significantly different by ANOVA are indicated by bars and asterisks as follows: *, *P* < 0.05; **, *P* < 0.01; ***, *P* < 0.001. Values that are not significantly different are indicated by bars labeled ns.

To determine whether expression of the *copB* and *mco* genes had an impact on bacterial growth under copper stress, we monitored the growth of cultures in TSB containing a concentration of copper below the MIC for all strains and mutants (4 mM [Fig. 2]). Strain 14-2553T(pSCBU) grew faster and to a higher OD_600_ in subinhibitory concentrations of copper than the same strain without the plasmid or the mutants deficient in *copB* or *mco* (Fig. 2A). The defect in growth was more pronounced for the *copB* mutant than for the *mco* mutant. In contrast, the growth profile of the 14-2533T CHC(pSCBU) mutant was identical to the growth profile of the wild-type strain carrying the plasmid (Fig. 2A), possibly due to the fact that CsoR does not fully repress *copB* and *mco* expression when they are carried on a replicating plasmid (2). There was no growth advantage observed for strains growing in TSB lacking copper (Fig. 2B) or when low (micromolar) concentrations of copper salts were added to the growth medium (data not shown). As a control, the growth of strain MRSA252 and the MRSA252 CsoR CHC mutant were compared in TSB containing a subinhibitory concentration of copper (4 mM [Fig. 2C]). Strain MRSA252 grew more quickly and reached a higher OD_600_ than the non-copper-responsive regulatory mutant MRSA252 CHC, but not in media without copper (Fig. 2D). Transcription of *copB* and *mco* in strains 14-2553T(pSCBU) and MRSA252 was quantified by reverse transcription-quantitative PCR (RT-qPCR) (Fig. 2E). An increase in the abundance of transcript was measured for strains carrying *mco* and *copB* genes in TSB cultures of 14-2553T(pSCBU) and MRSA252 supplemented with subinhibitory concentrations of CuCl_2_ (4 mM) compared to TSB without added copper, which confirmed that expression of *copB-mco* can be induced by copper. For strain MRSA252 CHC, no *copB* or *mco* expression was detected in bacteria grown in TSB supplemented with CuCl_2_ (4 mM), showing that this strain is unable to express the *copB*-*mco* operon in response to CuCl_2_. The expression of *copB* and *mco* in the 14-2533T CHC(pSCBU) mutant was induced to a much lesser extent by copper than in the parent strain 14-2533T(pSCBU), and the small increase was not statistically significant. RNA transcripts of *mco* or *copB* were not detected in their respective deletion mutants (Fig. 2E and Fig. S1), as expected. Inducible copper-dependent expression of the other gene was detected in each of the mutants, showing that the respective gene deletions had not obstructed transcription of the other gene in this operon from the P*_copB_* promoter (Fig. 2E).

### Copper hypertolerance genes increase S. aureus survival inside IFN-γ-activated macrophages.

Copper has previously been shown to be critical for the killing of bacteria following phagocytosis (7). In the presence of copper, activated macrophages upregulate expression of the copper importer, CTR1, and commence trafficking of the P-type ATPase ATP7A to the phagolysosomal membrane, which leads to an enhanced killing of intracellular bacteria (7, 10).

**FIG 3:**
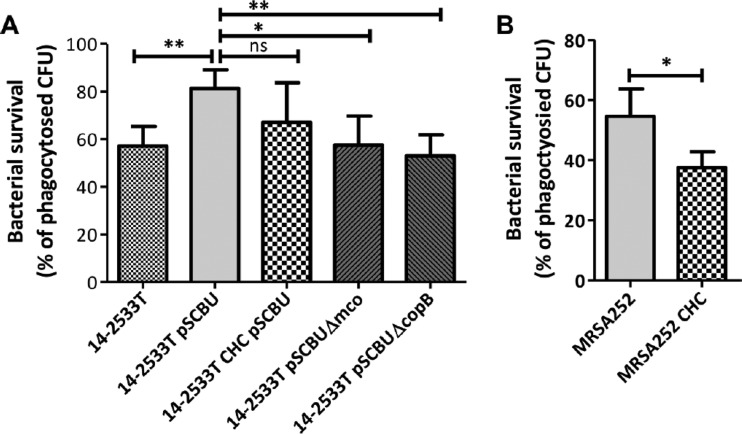
Hypertolerance to copper increases resistance of S. aureus to macrophage killing. Mouse macrophage cell line (RAW264.7) was suspended in DMEM supplemented with mouse IFN-γ (40 ng/ml) and CuSO_4_ (40 µM) and seeded in the wells of 24-well plates at 2 × 10^6^ cells per ml for 18 h at 37°C in 5% CO_2_. (A and B) S. aureus strain 14-2533T (A) or MRSA252 (B) and derivatives were grown overnight in RPMI 1640 and then inoculated into the wells at a multiplicity of infection (MOI) of 10 in DMEM allowing phagocytosis for 30 min followed by killing of extracellular bacteria with gentamicin/lysostaphin for 30 min. Macrophages were then lysed at this time point (time zero [T0]) and after 3 h of incubation (T3), and viable bacteria were counted to determine the levels of bacterial survival. The mutants expressing CsoR C41A/H66A/C70A (CHC) are indicated. Values are means plus SD from three independent experiments. Statistical significance is indicated as follows: **, *P* < 0.005; *, *P* < 0.05; ns, not significant.

To investigate whether bacterial tolerance to copper might influence the outcome for S. aureus following phagocytosis by macrophages, experiments were performed to quantify the survival of bacteria following phagocytosis. The murine macrophage cell line (RAW264.7) was activated with IFN-γ and treated with CuSO_4_ to induce expression of the relevant copper transporters (ATP7A and CTR1), which was confirmed using RT-qPCR (Fig. S2) (7, 27). IFN-γ-activated phages internalized the wild-type strain and mutants at similar levels (data not shown). However, 3 h after phagocytosis, intracellular levels of bacteria were significantly different in the strains. The 14-2533T(pSCBU) strain survived inside the macrophages at significantly higher levels than strain 14-2533T without the plasmid (Fig. 3A). Importantly, the copper-susceptible *copB* and *mco* mutants had a survival defect compared to their parent strain 14-2533T, suggesting that copper tolerance in S. aureus prevents killing by macrophages (Fig. 3A). The CsoR CHC mutant of 14-2533T(pSCBU) did not show a significant survival defect in macrophages (Fig. 3A), probably reflective of the fact that CsoR-regulated genes carried on plasmids are not efficiently repressed in this strain (2) (Fig. 2 ), thus, it behaves like the wild type. In contrast, the MRSA252 CsoR CHC mutant had a defect in macrophage survival (Fig. 3B).

10.1128/mBio.00550-18.2 FIG S2 IFN-γ- and copper-induced expression of copper transporters in murine macrophage cell line RAW267.4. RT-qPCR was performed using RNA isolated from macrophages untreated or treated with murine IFN-γ (40 ng/ml) and CuSO_4_ (40 μM). The ΔΔ*C_*T*_* method was used to determine the relative expression levels of genes encoding transporters and normalized to *gyrB*. Values are means plus SD from three independent experiments, with statistical significance determined by ANOVA and Dunnett’s posttest (**, *P <* 0.05). Download FIG S2, TIF file, 0.1 MB. Copyright © 2018 Zapotoczna et al. 2018 Zapotoczna et al. This content is distributed under the terms of the Creative Commons Attribution 4.0 International license.

**FIG 4:**
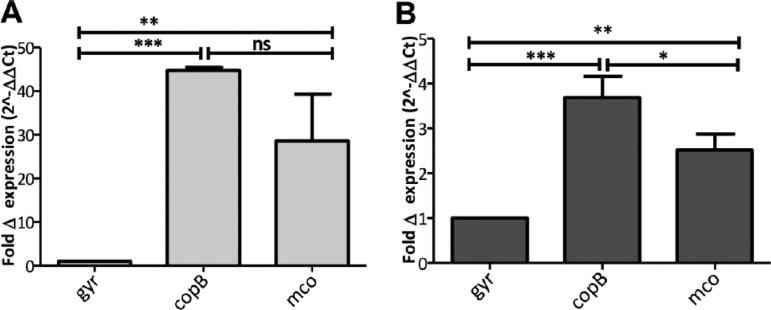
Intracellular expression of *copB* and *mco*. (A and B) Fold change in expression of *copB* and *mco* by wild-type S. aureus relative to CsoR CHC mutants of either S. aureus 14-2533T(pSCBU) (A) or MRSA252 (B). RAW264.7 macrophages were activated with IFN-γ (50 µg/ml) and copper chloride (40 µM) for 18 h. Infections of the macrophage monolayer were performed with S. aureus grown in RPMI 1640 at an MOI of 20. Extracellular bacteria were killed by treatment with gentamicin/lysostaphin following by washing of the monolayers with phosphate-buffered saline (PBS). Three hours postinfection, RNA was isolated from infected macrophages and for RT-qPCR. The ΔΔ*C_T_* method was used to determine the relative expression levels of the *mco* and *copB* genes in WT and CHC mutants normalized to *gyrB*. Values are means plus SD from three independent experiments. Statistical significance was determined by ANOVA and Bonferroni’s multiple-comparison posttest and indicated as follows: *, *P* < 0.5; **, P < 0.05; ***, *P* < 0.005; ns, not significant.

To determine whether the *copB* and *mco* genes are expressed by bacteria residing inside activated macrophages, RT-qPCR was performed using RNA obtained from intracellular bacteria at 3 h postinfection. The relative transcription levels were compared between the wild-type strains and their isogenic CsoR CHC mutants. The *copB* and *mco* genes were found to be 44- and 28-fold upregulated, respectively, in wild-type MRSA252 compared to MRSA252 CHC recovered from infected macrophages (Fig. 4A). This demonstrated that (i) *copB* and *mco* are expressed by S. aureus inside the macrophage and (ii) this expression is dependent on CsoR within immune cells (Fig. 4A). The same experiment was carried out with strain 14-2533T(pSCBU) and showed that *copB* and *mco* are expressed intracellularly in macrophages (Fig. 4B). However, the increase in expression of *copB* and *mco* was much less for 14-2533T(pSCBU) than in the MRSA252 strain (Fig. 4B), which is consistent with susceptibility results (Table 1 and Fig. 2), indicating a weaker transcriptional control of CsoR over the plasmid-borne genes compared to the genes carried on the chromosome of MRSA252.

### Copper hypertolerance genes increase survival of S. aureus in whole human blood.

**FIG 5:**
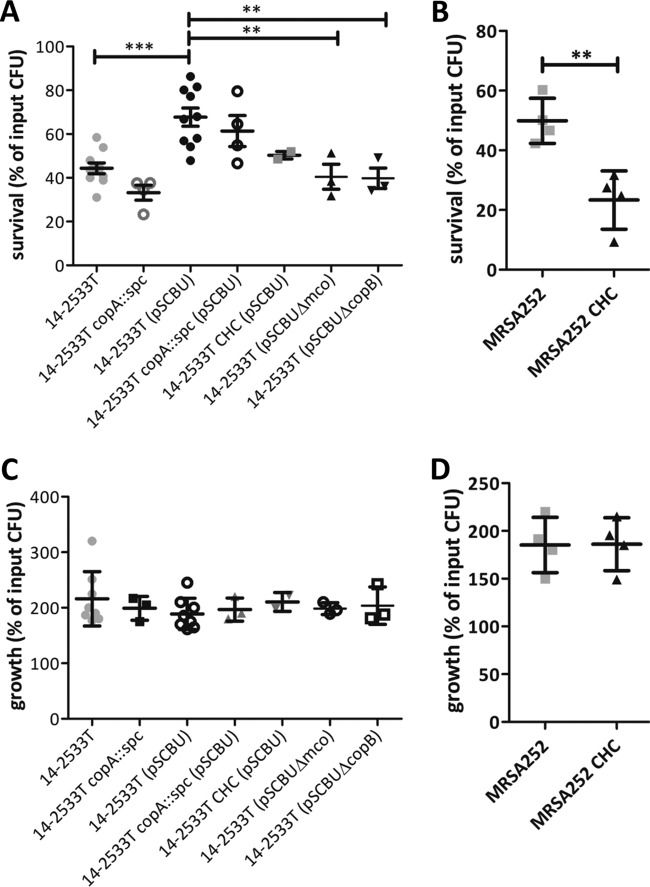
Increased survival of copper-hypertolerant S. aureus in human blood. S. aureus (ca. 1 × 10^4^ CFU/ml) strains were inoculated into freshly drawn human blood (A and B) or plasma (C and D) and incubated for 3 h at 37˚C. Viable counts were used to determine the numbers of bacteria in blood or plasma. The number of CFU after 3 h is expressed as a percentage of the original input CFU at 0 h. Horizontal lines represent the means ± SD from at least three independent experiments. Statistical significance was determined by ANOVA following Dunnett’s multiple-comparison test (A) or by an unpaired *t* test (B) and indicated as follows: for panel A, **, *P* < 0.01; ***, *P* < 0.001; for panel B, **, *P* = 0.0052.

To determine whether the enhanced ability of *copB-mco*-carrying strains to survive inside activated macrophages *in vitro* may be of relevance to infection of the human host, *ex vivo* infection studies were performed with whole human blood. Consistent with results obtained for intracellular survival within activated macrophages, copper-hypertolerant S. aureus 14-2533T(pSCBU) had an increased ability to survive in whole human blood compared to the 14-2533T strain without the plasmid (Fig. 5A). This protection from killing in blood was due to copper resistance genes, since the *mco* and *copB* mutants had a survival defect, similar to that of the plasmid-deficient 14-2533T strain (Fig. 5A). Protection from killing in blood could be attributed to resistance to phagocytic killing, since incubation in the cell-free plasma fraction of the same blood under the same conditions yielded similar values for the wild type and mutants (Fig. 5C). There was no significant difference in the survival of the *copA* mutants in blood (Fig. 5A), suggesting that *copB* and *mco*, but not *copA*, confer protection against cellular killing in human blood.

The CsoR CHC mutant of strain MRSA252 had a significant defect in survival in whole blood compared to the wild type but did not show a defect in growth in plasma (Fig. 5B and D). This showed that failure to derepress CsoR-regulated genes (Fig. 1) impaired the ability of S. aureus to survive in blood. Together, these results show the importance of copper hypertolerance for S. aureus to resist cellular killing in human blood.

### The *copB-mco* operon is carried by invasive S. aureus isolates and by strains belonging to CC22, CC30, and CC398.

The prevalence of the *copB-mco* operon was investigated by interrogating the whole-genome sequences (WGS) of 308 invasive S. aureus isolates (28) from hospitals across Europe. Mapping the *copB*-*mco* sequences against the WGS showed that this operon was present in 55 of the invasive isolates (17.9% [Fig. S3]). The *copB* and *mco* genes were carried by isolates from two major clonal complexes (CCs) within the population, clonal complex 22 (CC22) and CC30, and also a single CC8 isolate. All CC30 strains carried the *copB*-*mco* operon. The most prevalent sequence type (ST) in the CC30 population carrying *copB*-*mco* was sequence type 30 (ST30), but ST2868, ST36 (EMRSA-16), ST2858, ST2864, ST2879, ST39, ST1829, ST2862, ST2881, and ST34 isolates also carried the *copB*-*mco* operon. Among the CC22 strains, 50% were found to carry the operon, and all of them belonged to ST22 apart from one ST2877 isolate. In summary, *copB*-*mco* was found to be present in invasive S. aureus strains from across Europe but predominantly in isolates from two important clonal groups, CC22 and CC30 (28).

10.1128/mBio.00550-18.3 FIG S3 Distribution of the P2-hm plasmid, including the *copB*-*mco* locus, in S. aureus isolates from hospitals across Europe. The presence and absence of short-read data of S. aureus isolates against the P2-hm sequence (accession number NC_019009) by mapping using Burrows-Wheeler Aligner (H. Li and R. Durbin, Bioinformatics 25:1754–1760, 2009). The presence (red) and absence (blue) of mapped sequence reads is indicated. The annotation of the P2-hm plasmid is displayed at the top of the mapping block. Protein-coding sequences are colored according to their predicted function as follows: orange, hypothetical proteins; pink, transposon; red, plasmid replication; brown, pseudogene; green, membrane protein; white, lantibiotic; gray, miscellaneous function. Isolate mapping data are ordered according to the core single nucleotide polymorphism (SNP) maximum likelihood tree, generated using Fast Tree (M. N. Price, P. S. Dehal, and A. P. Arkin, Mol Biol Evol 26:1641–1650, 2009), displayed on the left. Download FIG S3, PDF file, 0.2 MB. Copyright © 2018 Zapotoczna et al. 2018 Zapotoczna et al. This content is distributed under the terms of the Creative Commons Attribution 4.0 International license.

To further explore the presence of *copB* homologs as well as related copper tolerance genes, we interrogated all publicly available S. aureus genomes (GenBank; *n* = 8,037). While a conserved *copA* was found universally in 99.9% of all genomes, *copB* homologs were the second most prevalent copper tolerance gene at ca. 34.4% of all the genomes. The *copB* and *mco* homologs were found mostly in CC22, CC30, and CC398 and only sporadically in other clonal complexes. To further characterize the distribution of genes in these three CCs, we constructed phylogenetic trees of each CC and mapped the presence and absence of each *copB* gene to each tree. Interestingly, the distributions of genes within each clade are strikingly different. For instance, CC30 genomes show a strong conservation of *copB* loci with very few predicted losses, whereas CC22 and CC398 have much more sporadic distributions that suggest multiple acquisitions and losses. This pattern could signal stronger, or more persistent, selection for *copB* loci in CC30 genomes compared to CC22 and CC398, where selection may be weaker or intermittent. We also found evidence of a more diverse context to the copper hypertolerance genes than the original context in which they were found, i.e., the *copB*-*mco* operon, with an additional putative lipoprotein-encoding gene *copL* (4, 29) frequently associated with the *copB*-*mco* operon in CC398 strains and less frequently in CC22 and CC30 strains. These data indicate that the *copB*-*mco* copper hypertolerance genes are widely distributed in CC22, CC30, and CC398 and imply the presence of selection pressure for hypertolerance to copper.

## DISCUSSION

The connection between gain of copper tolerance and increased virulence of several human pathogens has been reported over recent years. Here we demonstrate that S. aureus employs copper hypertolerance genes to resist macrophage killing and to survive in whole human blood. Presumably, better survival in human blood is due to an increased resistance to killing by the cellular component, because control experiments indicated that growth in blood plasma was not affected by copper resistance genes (Fig. 5). The increased resistance to phagocytic killing conferred by the *copB*-*mco* operon is likely to affect the virulence potential of the bacterium *in vivo* and may provide a selective advantage to the pathogen. Importantly, *copB* and *mco* were expressed within infected macrophages, and the expression of these genes was, at least partially, dependent on expression of copper-responsive CsoR (Fig. 4). This provides indirect evidence that the *copB*-*mco* operon is expressed intracellularly in macrophages, in response to copper. ATP7A-dependent copper transport into the macrophage phagosome is required for bactericidal activity (7). Since copper hypertolerance genes confer resistance to killing in a macrophage cell line, it is tempting to speculate that the protective effect of CopB and Mco is due to increased tolerance of S. aureus to copper within the phagosome. However, further investigation is needed to fully understand how CopB and Mco exert their effect in phagocytic cells.

**FIG 6:**
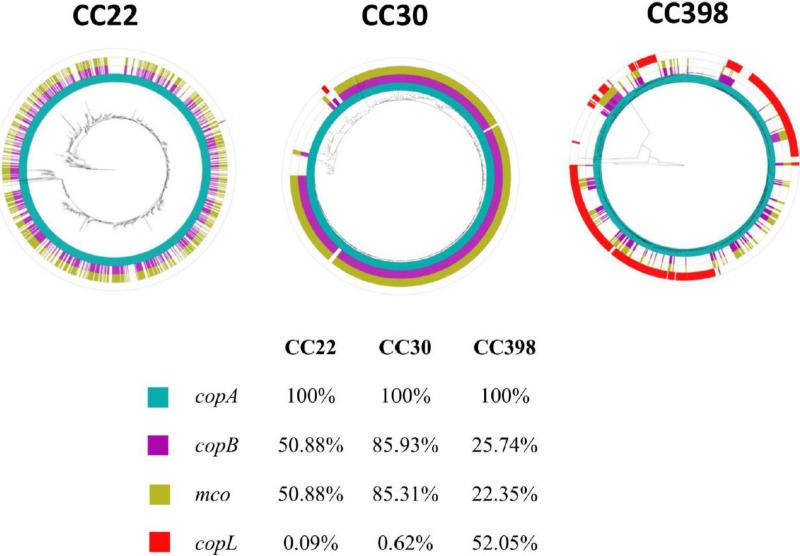
Maximum likelihood trees of CC22, CC30, and CC398 showing distribution of *copA*, *copB*, *mco*, and *copL* genes. Trees are rooted in the longest branch (CC22 to S. aureus 08 01492; CC398 to S. aureus SO1977; CC30 to S. aureus MRSA252). The four rings show the presence of *copA*, *copB*, *mco*, *copL* as blue, purple, yellow, and red, respectively.

By studying the genome sequences of a collection of invasive isolates obtained from hospitals across Europe, we determined the prevalence of *copB-mco* to be 17.9% of all isolates, emphasizing the clinical relevance of this locus (5, 28, 30). The *copB* and *mco* genes were carried by all isolates belonging to CC30, by 50% of isolates belonging to CC22, and by a single CC8 isolate. The plasmid carrying the *copB-mco* operon was also recently reported to be carried by 43 to 70% of bloodstream infection isolates of S. aureus (mostly CC22) from the United Kingdom and Ireland sampled between 2001 and 2010 (3). There is evidence of extensive loss and gain of the pSCBU (p2-hm) plasmid (3), highlighting the mobility of the *copB-mco* operon within populations of S. aureus. The global significance of copper resistance in S. aureus was further highlighted by the widespread presence of *copB* and *mco* in CC22, CC30, and CC398 strains (Fig. 6). Interestingly, CC398 is the most common CC found in European livestock. Previous studies have reported that 24.3% of livestock-associated MRSA carried the *copB* gene (31). The use of copper compounds as feed supplements in animal husbandry may be selecting for the carriage of copper resistance genes by MRSA (32). Copper hypertolerance in S. aureus is likely to have broader implications for human health, since the dominant clone of community-associated (CA)-MRSA in North America (USA300) and a closely related CA-MRSA clone found in South America (USA300-LV) both independently acquired a copper resistance locus as part of the arginine catabolic mobile element and the copper and mercury resistance element, respectively (4). In both cases, the copper resistance loci are adjacent to the staphylococcal cassette chromosome *mec* element (SCC*mec*).

Consistent with a previous report (2), our data show that the *copB-mco* operon mediates copper hypertolerance in S. aureus. Disruption of the *copB* or *mco* gene inhibited the growth of S. aureus in subinhibitory concentrations of copper, demonstrating that carriage of both of these genes provides a fitness advantage to S. aureus under copper stress. Therefore, it can be concluded that both CopB-mediated copper efflux and the activity of Mco play a role in protecting S. aureus from copper.

The CsoR repressor, which has been previously implicated in transcriptional regulation of *copA*-*copZ* and *copB*-*mco* (2), was shown here to control expression of *copA* and *copB*-*mco* in a copper-dependent manner by binding directly to the DNA sequence upstream of *copA* and *copB* but not of *mco* (Fig. 1). Inactivation of the Cu(I)-coordinating residues Cys41, His66, and Cys70 (CHC) disrupted copper-dependent derepression of CsoR-regulated genes. Although continued association of CsoR CHC with the *copA* and *copB* promoter DNA was confirmed by EMSA using recombinant proteins (Fig. 2), repression of the copper tolerance phenotype by the CsoR CHC variant was completely effective only in live bacteria with the chromosomally encoded *copB*-*mco* operon (strain MRSA252 CHC). In contrast, the CHC mutant of 14-2533T(pSCBU) did not completely lose the copper hypertolerance phenotype shown by the parent strain carrying a wild-type copy of the *csoR* gene (Table 1 and Fig. 2 and 4), due to the fact that CsoR does not fully repress *copB* expression from a plasmid (2). It may be that CsoR diffuses poorly to the pSCBU plasmid-located genes or that there are too few copies of the CsoR protein in the cell to fully repress *copB-mco* if the plasmid is present in more than one copy. Surprisingly, mutation of *copA* had no significant effect on copper tolerance phenotypes under the conditions used here to study *copB*-*mco* function. Our recent study conducted with S. aureus USA300 JE2 strain revealed that mutation of *copA* can influence expression of other copper-regulated genes elsewhere on the chromosome (29). A more extensive analysis of the transcriptome of S. aureus copper-hypertolerant strains will be needed to fully understand the phenotypes of *copA* mutants.

Horizontal gene transfer represents a major driving force in the evolution of S. aureus (33). This study provides important new insights into the contribution of MGE-carried copper hypertolerance genes to the resistance of S. aureus to innate immune defenses. Due to the potential for MGEs to transmit rapidly in populations of S. aureus, our study shows that the spread of copper hypertolerance genes could have important implications for the evolution of S. aureus as a pathogen.

## MATERIALS AND METHODS

### Bacterial strains and growth conditions.

S. aureus strains used in the study are listed in Table S1 in the supplemental material. Bacteria were grown on tryptic soy agar (TSA) plates or in liquid cultures in either tryptic soy broth (TSB) or RPMI 1640 at 37°C with shaking (200 rpm). To select for strains carrying pSCBU, TSA was supplemented with CdCl_2_ at 1 mM. Growth curves were obtained using microtiter plates in TSB containing copper salts (either CuCl_2_ or CuSO_4_). For macrophage and whole-blood survival assays, bacterial strains were cultured in RPMI 1640 in aerated 50-ml Falcon tubes at 37°C with shaking (200 rpm).

### Construction of mutations in plasmid-borne and chromosomally integrated *copB* and *mco* genes.

Plasmid pSCBU was extracted from strain SASCBU26 (34) and used to transform strain 14-2533T (Table S1). Mutations in S. aureus, including deletions in the native plasmid pSCBU (Table S1 and Fig. S1), were introduced using pIMAY (35). Plasmids with deletions of the copper tolerance genes, pSCBUΔ*mco* and pSCBUΔ*copB*, were isolated in the 14-2533T (clonal complex 22 [CC22]) background (Table S1 and Fig. S1). It was necessary to purify and reintroduce each validated mutated plasmid into a clean background in order to eliminate a mixed population containing a mutated and wild-type copy of this multicopy plasmid. Strain 14-2533T *copA*::*spc* was constructed by transduction of *copA*::*spc* (29) into strain 14-2533T using phage 85.

### Susceptibility testing.

MICs of soluble metal salts were determined by the standard broth microdilution method according to the guidelines by Clinical and Laboratory Standards Institute (CLSI). The lowest concentration of a compound showing no visible growth was recorded as the MIC.

### Production and purification of recombinant CsoR.

The wild-type *csoR* gene was amplified (Table S2) from S. aureus genomic DNA and cloned into pGEM-T (Promega). An internal NdeI site was mutated silently using QuikChange site-directed mutagenesis (Stratagene), and then *csoR* was subcloned into vector pET29a via NdeI/BamHI digestion and ligation. The *csoR* CHC mutant gene was amplified from the respective pIMAY construct. Constructs were confirmed by sequencing (GATC Biotech).

10.1128/mBio.00550-18.5 TABLE S2 Primers used in this study. Download Table S2, PDF file, 0.01 MB. Copyright © 2018 Zapotoczna et al. 2018 Zapotoczna et al. This content is distributed under the terms of the Creative Commons Attribution 4.0 International license.

Escherichia coli BL21(DE3) cells transformed with the resulting vector, pET29a-CsoR or pET29a-CsoR-CHC, were cultured in lysogeny broth (LB) at 37°C with orbital shaking at 180 rpm, and protein expression was induced at an optical density at 600 nm (OD_600_) of ∼0.6 by the addition of 1 mM isopropyl-β-d-1-thiogalactopyranoside (IPTG), followed by further incubation at 30°C for 5 h. Cells were harvested, washed, resuspended in 25 mM Tris (pH 7.5) and 15 mM dithiothreitol (DTT) containing protease inhibitor cocktail (Sigma), and lysed by sonication.

The supernatant was clarified by centrifugation and filtration and purified by anion-exchange chromatography on a 5-ml HiTrap Q HP column and an Akta purifier (GE Healthcare). Protein was eluted with a linear NaCl gradient (0 to 1 M NaCl), and CsoR-containing fractions (assessed by SDS-PAGE) subsequently concentrated on a 1-ml heparin column (GE Healthcare) eluted with 1 M NaCl. This fraction was incubated overnight at 4°C with 10 mM EDTA and 20 mM tris(2-carboxyethyl)phosphine (TCEP), before resolution on a Superdex 75 16/600 column in 25 mM 4-(2-hydroxyethyl)-1-piperazineethanesulfonic acid (HEPES) (pH 7.5), 200 mM NaCl, and 15 mM DTT.

### EMSA.

S. aureus MRSA252 genomic DNA was used to PCR amplify the putative promoter regions (i.e., the ∼200 bp upstream of the start codon) of *copA*, *copB*, and *mco* (Table S2), which were cloned into vector pGEM-T, confirmed by sequencing. The promoter fragments (plus ∼100 bp of flanking sequence from pGEM-T) were produced by PCR amplification from these pGEM-T constructs, plus a negative-control fragment containing only the pGEM-T sequences. These PCR products were purified and used in electrophoretic mobility shift assays (EMSAs).

EMSAs were performed by incubating fully reduced (as determined with Ellman’s reagent) recombinant CsoR variants (0 to 100 µM) with the respective promoter DNA plus the negative-control DNA (both 0.1 µM) in 20 mM HEPES (pH 7.0), 100 mM NaCl, 100 ng/µl poly(dI-dC) (Sigma), 1 mM DTT, 0.4 mg/ml bovine serum albumin (BSA) at room temperature for 30 min. All incubations were performed anaerobically inside an N_2_ atmosphere glove box ([O_2_] < 5 ppm) (Belle Technology), and Cu(I)-CsoR was prepared by anaerobically incubating protein for 10 min with 1 mol equivalent of Cu(I) prepared as previously described (36). After incubation, samples were resolved on 6% acrylamide (wt/vol) native PAGE for 60 to 80 min at 82 V and stained with 10% SYBR Safe solution (Invitrogen) for 20 min.

### Inductively coupled plasma mass spectrometry.

S. aureus 14-2533T and 14-2533T(pSCBU) bacteria were grown overnight in TSB and then subcultured into TSB or TSB supplemented with CuCl_2_ (4 mM) for 16 h. Samples were normalized to the same OD_600_, harvested by centrifugation, and washed twice in 50 mM Tris (pH 7.5), 100 mM NaCl, and 10 mM EDTA, followed by two washes in 50 mM Tris (pH 7.5) and 100 mM NaCl without EDTA. Washed pellets were stored at −20°C until use, then thawed, and digested with 65% (wt/vol) nitric acid (Merck) for 48 h. Digests were centrifuged at 21,000 × *g* for 20 min at 4°C, and the supernatants were analyzed using inductively coupled plasma mass spectrometry (Thermo x-series2). Samples were diluted 10-fold in 2% nitric acid containing 20 µg/liter platinum and indium as internal standards and analyzed (100 reads, 30-ms dwell, five channels, 0.02 atomic mass unit separation, each in triplicate) for ^55^Mn, ^65^Cu, ^66^Zn, ^114^In, and ^195^Pt in collision cell mode (3 ml/min 8% H_2_ in He collision gas), and metal concentrations were determined by comparison to matrix-matched elemental standard solutions (Merck).

### RNA extraction. (i) RNA isolation from S. aureus.

To isolate RNA from S. aureus, bacterial cultures were grown in 20 ml TSB with or without copper salts (as indicated) to an OD_600_ of ∼0.6. Cultures were suspended in phenol-ethanol (5:95) mixture and incubated on ice for 1 h before pelleting the cells by centrifugation. At this step, pellets were either stored at −70°C or subjected to total RNA extraction. To extract RNA, the pellet(s) was gently suspended in 1 ml of TRIzol following lysis using FastPrep lysing beads (three times, each time for 45 s, 2-min intervals on ice). Aqueous lysate was then mixed with chloroform (2:1) in Phase Lock Gel to separate the RNA-containing aqueous upper layer from the high-density organic lower phase. The upper phase was precipitated with isopropanol (1:1) following ultracentrifugation at top speed for 30 min. The pellet was washed with 70% (vol/vol) ethanol and centrifuged. Supernatant was removed, and the RNA pellet was dried.

### (ii) RNA isolation from macrophages.

RNA isolation from macrophages was performed using a modified TRIzol-based method. RAW264.7 cells were lysed directly in the culture dish by adding 12 ml of TRIzol per T-175 cm^2^ flask and scraping the cells. Chloroform was added to the suspension at 0.2 ml per 1 mM TRIzol reagent. Samples were immediately vortexed and incubated at room temperature for 2 to 3 min. Following centrifugation at 12,000 × *g* for 15 min at 4°C, the mixture separated into layers, and the upper aqueous layer was collected, precipitated with 0.5 ml isopropanol per 1 ml of TRIzol, incubated at room temperature for 10 min, and centrifuged at 12,000 × *g* for 10 min at 4°C. The RNA pellet obtained was washed once with 75% ethanol (adding at least 1 ml per 1 ml of TRIzol).

### (iii) RNA isolation from intracellular S. aureus.

To isolate RNA from intracellular S. aureus, a combination of the above methods was used. First, cells were infected in T-175 cm^2^ flasks following gentamicin/lysostaphin killing of extracellular bacteria and monolayer washing. The cells were then lysed with TRIzol as described above. Centrifugation at 4,000 × *g* for 20 min was performed to separate the bacteria into a pellet. RNA from the bacterium-containing pellet and macrophage RNA-containing suspension was extracted by the respective methods.

All air-dried pellets were dissolved in RNase-free molecular-grade water, and their stability and purity were checked by gel electrophoresis. The concentrations were determined using a Thermo Fisher Scientific NanoDrop spectrophotometer.

### RT-qPCR.

RNA was digested by DNase I treatment (Qiagen) according to the manufacturer’s instructions and quantified using a NanoDrop spectrophotometer, and the integrity of RNA was assessed by electrophoresis. RNA was reverse transcribed to cDNA using High Capacity RNA-to-cDNA kit (Applied Biosystems). Reverse transcription-quantitative PCR (RT-qPCR) was performed using the Power SYBR green PCR master mix (Applied Biosystems). The relative levels of gene expression in the treated cells and the nontreated controls were calculated by relative quantification using *gyrB* as the reference gene and using the primers in Table S2. All samples were amplified in triplicate, and data analysis was conducted using StepOne software (Applied Biosystems).

Genomic DNA was isolated from cultured macrophages as described previously (http://cancer.ucsf.edu/_docs/cores/array/protocols/dna_cell_culture.pdf). The isolated DNA was used as the template to generate a standard curve.

### Macrophage survival assays.

A murine macrophage cell line (RAW264.7) was cultured in Dulbecco modified Eagle medium (DMEM) containing 10% (vol/vol) fetal bovine serum (FBS). To generate monolayers, 2 × 10^6^ cells per ml were seeded in the wells of 24-well plates (500 µl per well) and incubated for 24 h in serum-free DMEM supplemented with CuSO_4_ (40 µM) and mouse interferon gamma (IFN-γ) (50 µg/ml) for 18 h at 37°C and 5% CO_2_. Immediately before the infection, RAW264.7 monolayers were washed with ice-cold DMEM alone. S. aureus strains were cultured in RPMI 1640. Immediately before the experiment, bacteria were washed twice with DMEM and adjusted to an OD_600_ of 0.05 (ca. 2 × 10^7^ of CFU per ml) in DMEM and inoculated into the monolayers for 30 min. The monolayers were subsequently washed, and extracellular bacteria were killed by treatment with gentamicin (200 µg/ml) and lysostaphin (100 µg/ml) for 30 min. Monolayers were then washed and lysed with ice-cold water at time zero (T0) and after additional 3 h of incubation (T3) to determine the survival rates (CFU per milliliter). Lysates were plated on agar, and CFU were counted to determine the numbers of viable bacteria.

### Human blood survival assays.

The quantification of S. aureus in human blood was performed by the method of O’Halloran et al. (37). Briefly, S. aureus variants were grown in RPMI 1640 to stationary phase and diluted in RPMI 1640, 25 µl (containing ca. 1 × 10^4^ CFU/ml) was added to 475-ml fresh blood samples obtained from healthy human volunteers, and blood samples were treated with 50 mg/ml of hirudin anticoagulant (Refludan; Pharmion). The tubes were incubated at 37°C with gentle rocking, and after 3 h, serial dilutions were plated to determine the CFU/milliliter of viable bacteria. In parallel, an equal inoculum was incubated with cell-free plasma derived from the same donor’s blood. Bacterial numbers in plasma were quantified (CFU/milliliter) at the 3-h time point, and percent survival of the original inoculum was determined. Ethical approval for the use of human blood was obtained from the Trinity College Dublin Faculty of Health Sciences ethics committee.

### Phylogenetic matrix construction and gene presence or absence.

All preassembled genomes from public databases for CC22, CC30, and CC398 (*n* = 1,075, 320, and 707, respectively) were used for whole-genome alignment with reference to the S. aureus N315 genome, using the NUCmer and show-snps utilities of MUMmer (http://mummer.sourceforge.net) (38). The S. aureus genomes were assigned sequence types (STs) and CCs by the S. aureus multilocus sequence type (MLST) typing scheme https://pubmlst.org/saureus/ site at the University of Oxford (39) using the MLST typing perl script v. 2.9 for contigs (https://github.com/tseemann/mlst), and thereafter membership in each CC was determined by clade membership in a large (*n* = 8,037; unpublished data) S. aureus data set composed of all publicly available preassembled genomes. All regions from the reference genome annotated as mobile genetic elements were excluded. We also applied a mask that excluded repetitive sequences from the reference genome that were >80% identical over at least 100 nucleotides to other genomic loci, based on pairwise MegaBLAST-based analysis (40). For each CC, a maximum likelihood phylogeny was constructed with RAxML v8.2.11 (41) using an ascertainment bias correction and the general time-reversible (GTR) substitution model (42) accounting for among-site rate heterogeneity using the Γ distribution and four rate categories (43) (ASC_GTRGAMMA model) for 100 individual searches with maximum parsimony random-addition starting trees. Node support was evaluated with 100 nonparametric bootstrap pseudoreplicates (44).

We used the *copA*, *copB*, and *copL* genes from strain TCH1516 and *mco* from strain CA12 to search for closely related genes in the genus *Staphylococcus* in GenBank (wgs and nr databases, 9,222 genomes as of 16 August 2017) using BLAST (tblastx with a cutoff value of 1e−130 for *copA*, *copB*, and *mco* and 1e−90 for *copL*) (45). The four genes were mapped to the three trees as high-quality circular representations using GraPhlAn software tool (https://bitbucket.org/nsegata/graphlan/). The richness of the color shows the percentage similarity with the seed sequence used.

### Statistics.

The data presented in this study are means ± standard deviations (SD) from three experiments unless stated otherwise. Statistical significance was assessed by two-way analysis of variance (ANOVA) and indicated in the figure legends.
