# Supplementary material for: Mobile-Genetic-Element-Encoded Hypertolerance to Copper Protects Staphylococcus aureus from Killing by Host Phagocytes
Source: mBio. 2018 Oct 16;9(5):e00550-18. doi: 10.1128/mBio.00550-18 (PMC6191537; doi:10.1128/mBio.00550-18)
Supplement: TABLE S1 [file mbo005184100st1.docx]

| Table S1. Strains used in this study | | |
| --- | --- | --- |
| Strains | Description | Source |
| 14-2533T | CC22 MRSA | Scottish Microbiology Reference Laboratories, Glasgow |
| 14-2533T CHC | 14-2533T mutant carrying the CsoR C41A/H66A/C70A variant | This study |
| 14-2533T *copA*:: *spc* | 14-2533T *copA* mutant created by tranducing the spectinomycin resistance (*aad9*) marked *copA* mutation from strain JE2 (29) into 14-2533T | This study |
| 14-2533T (pSCBU) | 14-2533T carrying plasmid pSCBU encoding *copB* and *mco* genes | This study |
| 14-2533T CHC (pSCBU) | 14-2533T CHC transformed with plasmid pSCBU | This study |
| 14-2533T (pSCBUΔ*mco*) | 14-2533T carrying a deletion of the *mco* gene on plasmid pSCBU | This study |
| 14-2533T (pSCBUΔ*copB*) | 14-2533T carrying a deletion of the *copB* gene on plasmid pSCBU | This study |
| 14-2533T *copA*::*spc* (pSCBU) | 14-2533T *copA*:: *spc* transformed with plasmid pSCBU | This study |
| SASCBU26 | CC22 MRSA. Source of pSCBU plasmid. | (34) |
| MRSA252 | CC30 MRSA. Carries the *copB*-*mco* locus integrated into the chromosome. | (5) |
| MRSA252 CHC | MRSA252 expressing the CsoR CHC variant (CsoR C41A/H66A/C70A) | This study |
| *E. coli* BL21(DE3) pET29a-CsoR | BL21(DE3) carrying the full length *csoR* gene cloned into pET29a | This study |
| *E. coli* BL21(DE3) pET29a-CsoR-CHC | BL21(DE3) transformed with pET29a carrying DNA encoding the CHC variant of CsoR | This study |
