# Supplementary figures and images for: Mobile-Genetic-Element-Encoded Hypertolerance to Copper Protects Staphylococcus aureus from Killing by Host Phagocytes
Source: mBio. 2018 Oct 16;9(5):e00550-18. doi: 10.1128/mBio.00550-18 (PMC6191537; doi:10.1128/mBio.00550-18)

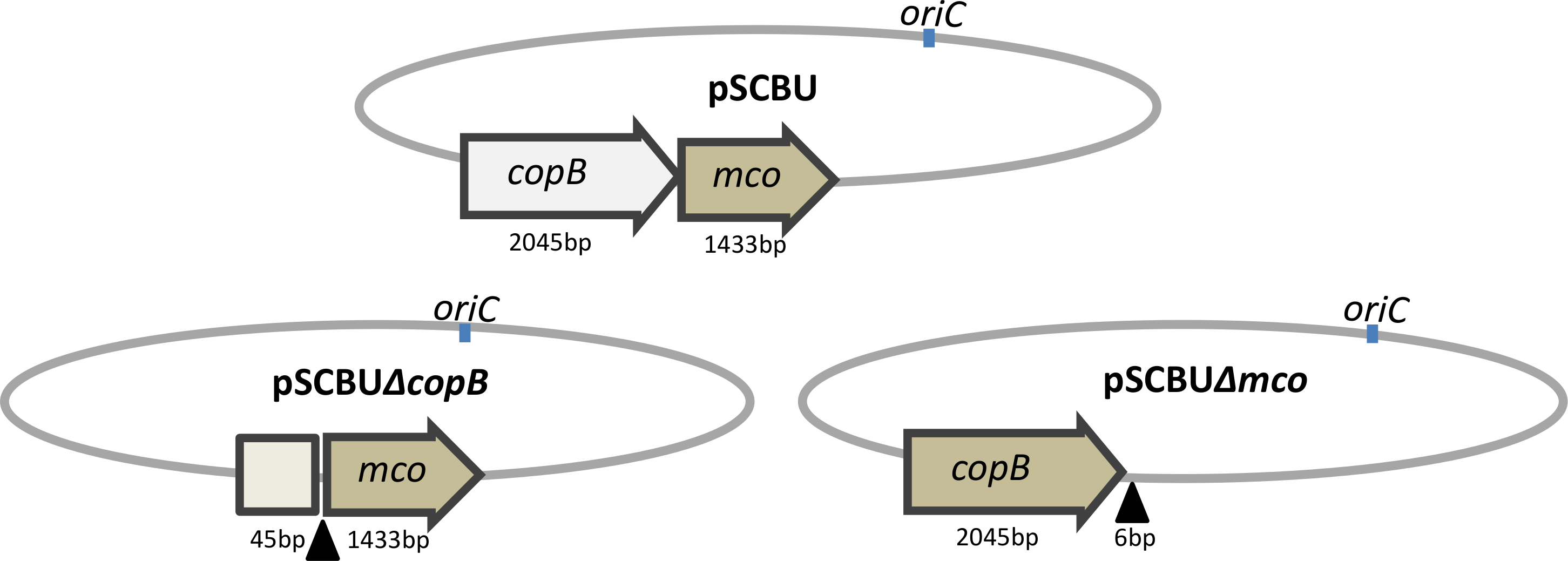

Supplement: FIG S1 [file mbo005184100sf1.tif]

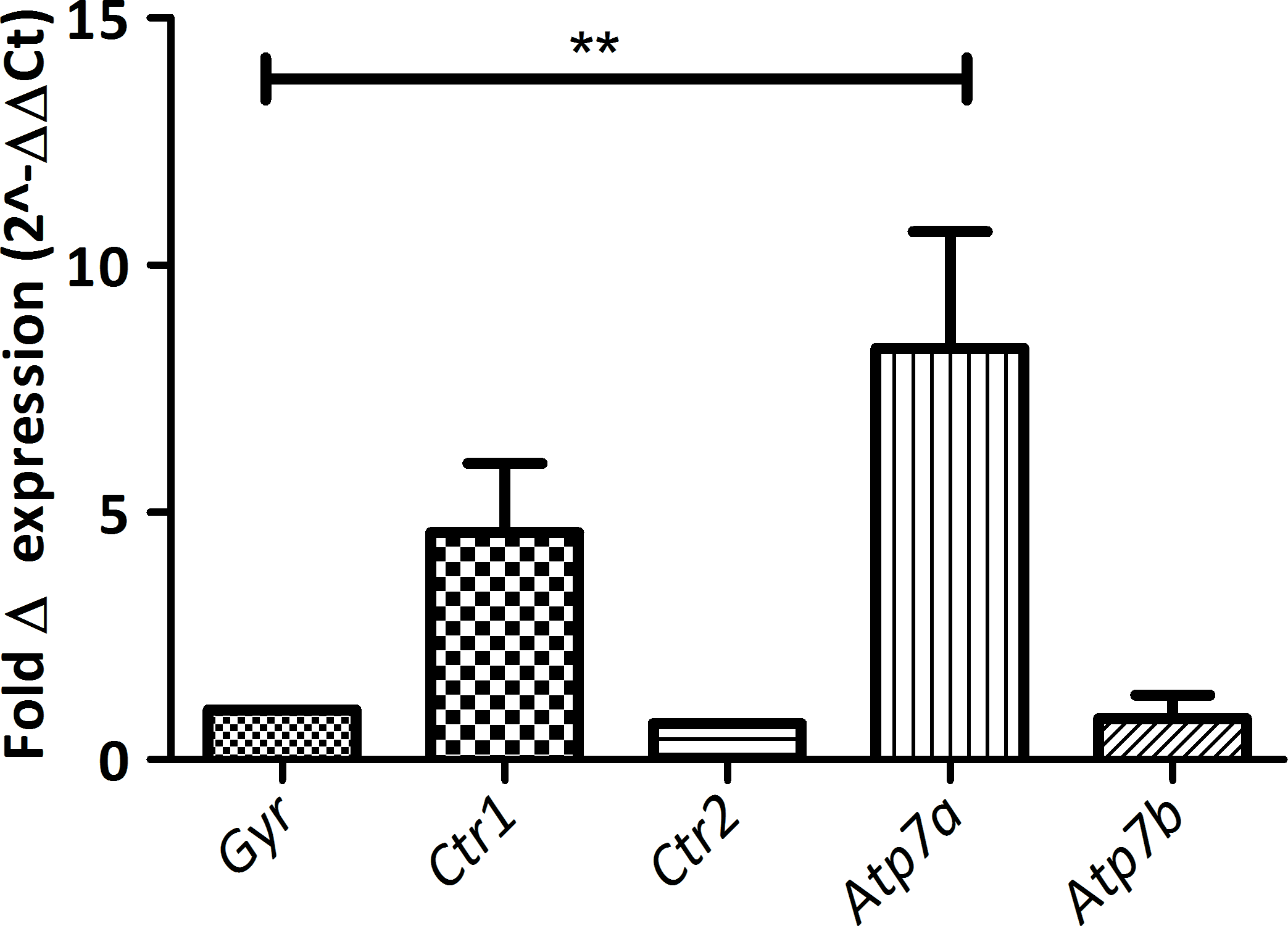

Supplement: FIG S2 [file mbo005184100sf2.tif]

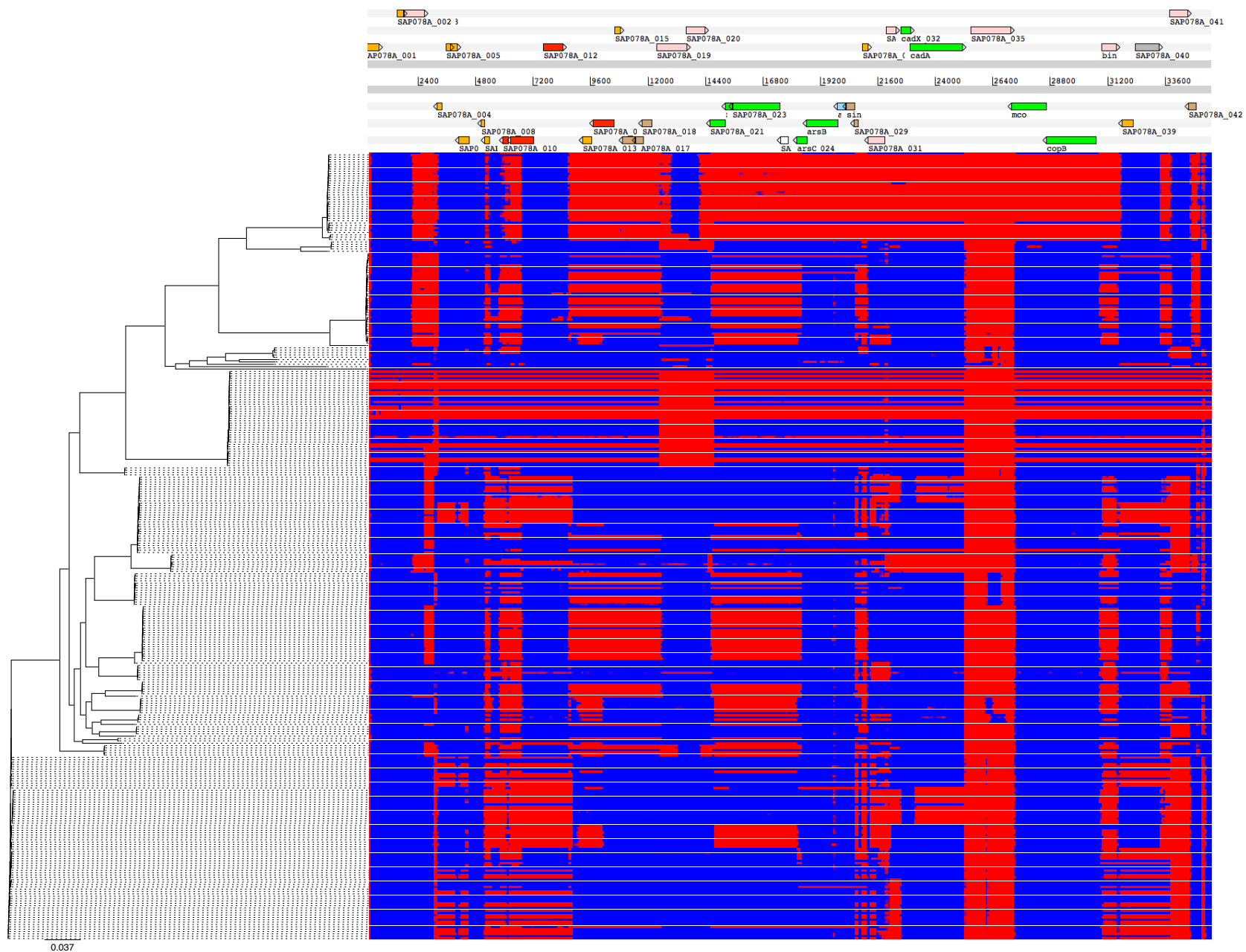

Supplement: FIG S3 [file mbo005184100sf3.pdf]
