# Supplementary material for: Mobile-Genetic-Element-Encoded Hypertolerance to Copper Protects Staphylococcus aureus from Killing by Host Phagocytes
Source: mBio. 2018 Oct 16;9(5):e00550-18. doi: 10.1128/mBio.00550-18 (PMC6191537; doi:10.1128/mBio.00550-18)
Supplement: TABLE S2 [file mbo005184100st2.pdf]

**Table S2 Primers used in the study**	
**EMSA primers**	
CsoR _F	GGGCATATGACTGAACAAGATAATGC
CsoR_R	GGGGGATCCTTAGTCTTTAATCAATTTTTGAA
CsoR _NdeI_F	GATAAAGTTATTAGAACAACACATGAAAAGTTGTATTATGAATAAAG
CsoR_NdeI_R	CTTTATT CATAATACAACTTTTCATGTGTTGTTCTAATAACTTTATC
PcopA _F	GGGGGATCCATTTCTTTTAAGTCACCTAAG
PcopA_R	GGGGATCCCACATTATTGCCTCCCTGT
PcopB_F	GGGGGATCCTTGTAATCCTGTTGAAACTCTTG
PcopB_R	GGGGGATCCCATATACGGGATAGGGGTATA T
Pmco_F	GGGGGATCCCTCGTTAAAAGTAATCCTTCTG
Pmco_R	GGGGGATCCCATTATATTTACCTCTTTTA
**qPCR primers**	
gyrB_F	ACCATAATGTAGCAGCCTCTTG
gyrB_R	GGCATGGGTTAGAAATAGATGGA
mco_F	ACCATAATGTAGCAGCCTCTTG
mco_R	GGCATGGGTTAGAAATAGATGGA
copB_F	GATTCCGTCTGTGGGTATACTTT
copB_R	CTGCAGTGAAGCTGATTGATAAC
Gadph_F	AGATCCCTCCAAAATCAAGTGG
Gadph_R	GGCAGAGATGATGACCCTTT
atp7a_F	TCAAGGAAAACAGTCAAGAGGA
atp7a_R	CGATGGGCAGGAAAACTC
atp7b_F	ACA TGG TTG GGA TAC CTA TTG C<br>TTG AGC TGA AGA GAC GAG AGC
atp7b_R	TTGAGCTGAAGAGACGAGAGC
ctr1_F	GAACCACACGGACGACAAC<br>AAGGTCATAGGCATCATCATCA
ctr1_R	AAGGTCATAGGCATCATCATCA
ctr2_F	ACAGGCATGGCTCTCTCAGT
ctr2_R	GAGGGTAGGCTGGCCTATAGGCT
